# Effect of Moxibustion on Inflammatory Cytokines in Animals with Rheumatoid Arthritis: A Systematic Review and Meta-Analysis

**DOI:** 10.1155/2020/6108619

**Published:** 2020-09-08

**Authors:** Yu-mei Zhong, Bo- Cheng, Lin-lin Zhang, Wen-ting Lu, Ya-nan Shang, Hai-yan Zhou

**Affiliations:** ^1^Acupuncture and Tuina School, Chengdu University of Traditional Chinese Medicine, Chengdu 610075, China; ^2^No. 1 Orthopedics Hospital of Chengdu, Chengdu 610015, China

## Abstract

**Background:**

This study aims to systematically evaluate the effect of moxibustion on the level of inflammatory cytokines in animal models with rheumatoid arthritis (RA) and to provide evidence for the clinical application of moxibustion to the treatment of RA and related basic researches.

**Methods:**

The databases employed in this study include PubMed, EMBASE, Web of Science, China National Knowledge Infrastructure (CNKI), Chinese Science and Technology Periodical Database (VIP), SinoMed, and Wanfang Data Information Site. The retrieval time was from the establishment of these databases to March 2020. The reviewers made use of the CAMARADES 10-item checklist to evaluate the quality of each included study. The inflammatory cytokines were considered as the outcome measure. The Revman 5.3 software was used to conduct meta-analysis on the outcome indicators of the studies included.

**Results:**

A total of 648 articles were retrieved and 18 animal experiments were included in this study. The quality scores of the studies ranged from two to eight with a mean of 5.8. Compared with the effect of the control group, moxibustion reduced the expression of TNF-*α* (SMD 2.95, 95% CI: 1.99–3.92, *P* < 0.00001), IL-1*β* (SMD 4.10, 95% CI: 2.37–5.84, *P* < 0.00001), IFN-*γ* (MD 25, 95% CI: 16.17–33.82, *P* < 0.00001), IL-6 (MD 11.83, 95% CI: 6.22–17.44, *P* < 0.0001), and IL-17 (MD 99.3, 95% CI: 86.83–111.76, *P* < 0.00001). At the same time, the level of IL-2 (SMD 8.89, 95% CI: 0.93–16.86, *P*=0.03), IL-4 (MD 1.79, 95% CI: 0.26–3.32, *P*=0.02), and IL-10 (MD 5.93, 95% CI: 1.37–10.49, *P*=0.01) increased after moxibustion treatment. Asymmetric funnel plots indicated that there was publication bias.

**Conclusion:**

The findings of the present review indicate that moxibustion can protect the synovium of joint in animal models with RA by upregulation of the level of anti-inflammatory cytokines and downregulation of the level of proinflammatory cytokines. Moxibustion has the potential to relieve inflammation of RA.

## 1. Introduction

Rheumatoid arthritis (RA) is an autoimmune disease that starts with the inflammation of synovium. The main symptoms of RA include pain, swelling, and stiffness of the joint. With the progression of the disease, cartilage destruction and bone erosion may occur, eventually leading to severe disability. The occurrence of RA is closely related to autoimmune disorders. Previous studies have revealed that many factors including gene, environment, hormone, and infection are involved in the persistent immunological response [1]. In addition, people with RA have an increased risk of cardiovascular disease, fragility fracture, and venous thromboembolism [2–4]. The incidence of RA varies from country to country and region to region, ranging from 0.5% to 1% in adults [5]. It is estimated that there are over 19 million prevalent cases of RA globally, which increased by 7.4% from 1990 to 2017 [6]. Global Burden of Diseases 2017 found that the crude disability adjusted life years rate increased by 25% during 1990–2017 [7]. The pain and joint dysfunction caused by RA urgently need an effective treatment to alleviate the inflammatory reaction and delay the progression of the disease. Among the RA therapeutic strategies, drug treatment is the most prominent one. However, both anti-inflammatory and biological agents have side effects (such as cardiovascular and gastrointestinal lineages complications) and patients treated with them may develop drug resistance [8, 9]. Therefore, it is of great value to seek a treatment that has significant clinical effect and low recurrence rate without side effects.

Moxibustion is an external therapy based on the theory of traditional Chinese medicine (TCM). As a complementary therapy, its effective mechanism may be associated with the thermal effects, radiation effects, and pharmacological actions produced by the burning of moxa and the influence of meridians and acupoints. The theory of TCM holds that moxibustion can invigorate Qi and stimulate blood circulation, warm meridians, and regulate Yin and Yang [10]. Accumulate studies demonstrate that moxibustion plays an important role in anti-inflammatory and pain relief, especially for the treatment of musculoskeletal diseases, such as primary osteoporosis [11], cervical spondylosis [12], and knee osteoarthritis [13]. Studies found that the therapeutic effects of moxibustion seem to be related to anti-inflammatory and immune regulation. In recent years, a large number of studies, especially animal experiment studies, have illustrated the anti-inflammatory effects of moxibustion for RA.

Reviews based on animal research will be beneficial to the design of animal experiment in the future and provide a basis for clinical studies. Furthermore, animal experiment provides important data and theoretical support for the potential mechanism of moxibustion therapy for RA. Therefore, the current study conducted a systematic review and meta-analysis to analyze the effect of moxibustion on inflammatory cytokines in animal models of RA to provide guidance for future animal experiments and clinical trials.

## 2. Methods

### 2.1. Registration

Following the preferred reporting items for systematic reviews and meta-analyses (PRISMA) guidelines, the manuscript has been registered in PROSPERO (No. CRD 42020171320).

### 2.2. Search Strategy

Four Chinese databases (CNKI, SinoMed, VIP, and Wanfang) and three English databases (PubMed, EMBASE, and Web of Science) were retrieved. The retrieval time was from the establishment of these databases to March 2020. The languages used in the studies were limited to Chinese and English. Search terms consisted of two aspects: subject (rheumatoid arthritis) and intervention (moxibustion and other related terms).

### 2.3. Inclusion Criteria

The inclusion criteria are as follows:

1. subjects: animal models with rheumatoid arthritis,
2. intervention: the therapy was moxibustion only and the forms of moxibustion were seed-sized moxa cone, suspension moxibustion, mild moxibustion, and so on,
3. outcome: inflammatory cytokines including tumor necrosis factor-*α* (TNF-*α*), interferon-*γ* (IFN-*γ*), interleukin-1*β* (IL-1*β*), interleukin-2 (IL-2), interleukin-4 (IL-4), interleukin-6 (IL-6), interleukin-10 (IL-10), and interleukin-17 (IL-17) were used to evaluate the anti-inflammatory effects of moxibustion,
4. language: Chinese or English.

### 2.4. Exclusion Criteria

1. Studies conducted in vitro or in humans
2. Moxibustion therapies combined with other therapies such as Chinese herbal medicine or Western medicine
3. The control group not treated with purified water injection, saline injection, or with no treatment
4. Studies without a separate control group
5. Duplicated publications, reviews, meta-analysis, or case reports; studies with abstract only; studies without extractable data

### 2.5. Study Selection and Data Extraction

One researcher (YMZ) retrieved the relevant databases according to the search strategy and made a list of all the records. Two evaluators (YNS and WTL) independently assessed the articles based on the inclusion and exclusion criteria. Firstly, the evaluators screened the title and abstract of the articles to exclude the obviously irrelevant literature. Secondly, the reviewers scanned the full text of these selected articles that met the previously mentioned criteria. If there was a disagreement, it would be resolved with negotiation between two researchers (YNS and WTL) or confirmed by the third researcher (LLZ).

Two researchers (YNS and WTL) extracted data from the selected studies. The contents of the data extraction mainly included the following: (1) general information: the first author, the year of publication, and nationality; (2) rat information: the number of rats in the experimental group and control group, gender, species, weight of rats, and modeling method; (3) treatment information: types of moxibustion, acupoint selection, duration, and period of treatment; (4) results: TNF-*α*, IFN-*γ*, IL-1*β*, IL-2, IL-4, IL-6, IL-10, and IL-17. When the data in the selected studies was presented as graph, the researchers would try to contact the author to obtain detailed data. If the author had no response after three times of e-mail contact, the study would be excluded since it lacked key information. The third reviewer would make a final decision on any divergence regarding the outcome data.

### 2.6. Quality Assessment

CAMARADES 10-item checklist was used to assess the methodological quality of the studies included [14]. The checklist included (1) peer-reviewed publication; (2) control of temperature; (3) random allocation to treatment or control; (4) blinded induction of model; (5) blinded assessment of outcome; (6) use of anesthetic without significant intrinsic neuroprotective activity; (7) appropriate animal model; (8) sample size calculation; (9) compliance with animal welfare regulations; (10) statement of potential conflict of interests.

Each study was given a comprehensive quality score out of a possible total of 10 points and the group median was calculated. Two reviewers (YNS and WTL) independently extracted data and assessed the quality of each study. Discrepancies were resolved by the third reviewer.

### 2.7. Statistical Analysis

Meta-analysis and statistical calculations were performed with the assistance of RevMan 5.3 software. The data of inflammatory cytokines were considered as continuous data. Mean difference (MD) would be employed to estimate the combined effect sizes of the indicators when the outcomes among different studies were tested with the same way. If the measurement unit of the outcome indicator was different, the standardized mean difference (SMD) would be given to estimate the combined effect sizes. The confidence interval (CI) was established at 95%. The *I*^2^ statistic was given to assess the heterogeneity among these studies. If *I*^2^ < 50%, it indicated that the heterogeneity among the studies included was low and a fixed effect model would be used. Otherwise, a random effect model would be performed. When significant heterogeneity existed, the sensitivity analyses and subgroup analysis would be conducted according to the moxibustion methods, animal species, animal weight, animal gender, and modeling methods. Funnel plot was used to evaluate the publication bias. When *P* value was less than 0.05, the outcome would be statistically significant.

## 3. Results

### 3.1. Study Inclusion

**Figure 1:**
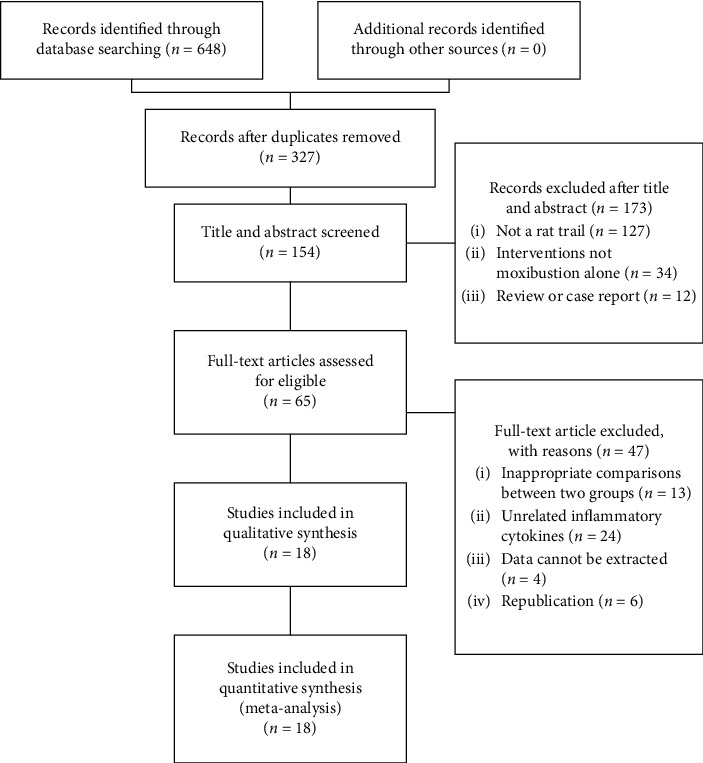
Flow diagram of the study selection process.

Initially, 648 studies were retrieved from seven databases. After removing duplicates, there were 327 articles left. According to the titles and abstracts of these records, the reviewers excluded 173 articles, because some studies were not about animal experiments, some studies were conducted in vitro, and some were reviews or case reports, while others were related to moxibustion therapies combined with other therapies and so on. After a full-text scanning, 47 articles were excluded. Eventually, 18 studies were selected [15–32]. The flow diagram of the study selection process is shown in Figure 1.

### 3.2. Study Characteristics

The eighteen included studies involved 461 rats, 231 of which were in the moxibustion group and 230 of which were in the control group. Among them, seventeen studies mentioned the weight of the rats, ranging from 110 to 250 g. The age of rats was different and was only stated specifically in four studies [18, 19, 25, 28]. It ranged from seven weeks old to sixteen weeks old. Four studies described rats as adult without offering the specific age of rats [16, 20, 26, 30]. All studies stated the gender of animals. Ten studies selected males [15–18, 21, 22, 24, 25, 27, 28] and eight studies had equal numbers of males and females [19, 20, 23, 26, 29–32]. Different inflammatory cytokines were used as outcome data in these studies: eleven studies reported data as TNF-*α* [15–25], three studies reported data as IFN-*γ* [23, 30, 32], eight studies reported data as IL-1*β* [15–18, 23, 25–27], three studies reported data as IL-2 [25, 28, 29], three studies reported data as IL-4 [23, 27, 30], three studies reported data as IL-6 [21, 26, 31], two studies reported data as IL-10 [30, 32], and two studies reported data as IL-17 [16, 24]. Two species of rats were used in the eighteen studies: thirteen studies used Sprague-Dawley (SD) rats [15–21, 26, 27, 29–32] and five studies used Wistar rats [22–25, 28]. Thirteen out of the eighteen studies were CFA models [16, 19–21, 23, 25–32]. Four studies were CIA models [15, 17, 18, 24]. CFA combined with CIA model was selected in one study [22]. The basic characteristics of the included studies are shown in Table 1.

### 3.3. Description of Moxibustion

Various moxibustion techniques were used in terms of the selection of acupoints, manipulation, and stimulation methods. The frequently used acupoints are Zusanli (ST36) and Shenshu (BL23), which have been used in fourteen studies [15–18, 20–27, 31, 32]. One study used governor meridian [19]. One study used Guanyuan (CV4) and Mingmen (GV4) [29]. One study used BL23 and behind Houzusanli (ST36) [28]. The acupoints involved in these studies also included Yanglingquan (GB34), Yinlingquan (SP9), or the combination of the previously mentioned points. The frequency of moxibustion was once a day. Rats received moxibustion treatment from 10 to 30 min per session. The period of moxibustion ranged from 12 to 25 days and most studies were 21 days. Eight studies chose seed-sized moxa cone as the method of stimulation [16, 21, 22, 26–28, 30–32], eight studies used suspension moxibustion [15, 17, 18, 20, 22–25], and the other two studies applied Tian moxibustion [29] and Tai-yi moxibustion, respectively [19]. Details of the moxibustion treatment are listed in Table 1.

### 3.4. Control Interventions

To better evaluate the anti-inflammatory effects of moxibustion, the current study excluded studies using therapies including western medicine, traditional Chinese medicine, or acupuncture for the control group and chose studies employing therapies like purified water injection, saline injection, or no treatment for the control group. All literature included set no treatment as control interventions.

### 3.5. Study Quality Assessment

The quality score of the included studies ranged from two to eight. More specifically, one study scored two, two studies scored three, five studies scored four, four studies scored seven, and six studies scored eight. Fourteen studies were published in peer-reviewed journal [16–21, 23–29, 32], but four studies were not because they were master's or doctoral thesis [15, 22, 30, 31]. Eleven studies elaborated the control of temperature [17, 18, 20, 23–26, 28–30, 32]. All studies randomly allocated rats to the control group and the treatment group and adopted appropriate animal models. Blinded induction of model and blinded assessment of outcome were stated, respectively, in four [17, 18, 23, 24] and five studies [20, 26, 28, 31, 32]. Eleven studies reported the use of anesthetic without significant intrinsic neuroprotective activity [16, 17, 20, 22, 23, 25, 26, 28, 29, 31, 32]. No study mentioned sample size calculation. Compliance with animal welfare regulations was stated in eleven studies [17, 18, 20, 22–26, 28,29, 32]. Twelve studies reported the potential conflict of interests [17, 18, 20, 21, 23–29, 32]. The basic characteristics of the study quality are presented in Table 2.

### 3.6. Outcomes Analysis

#### 3.6.1. Proinflammatory Cytokines Outcomes Analysis

**Figure 2:**
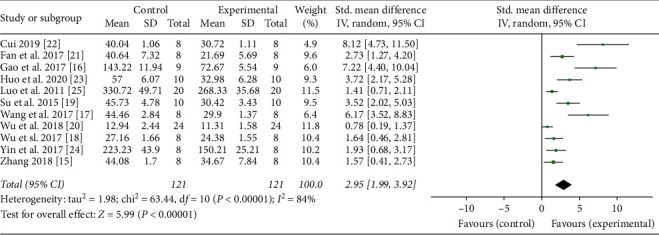
Forest plot showed that the level of TNF-*α* decreased with moxibustion therapy.

**Figure 3:**
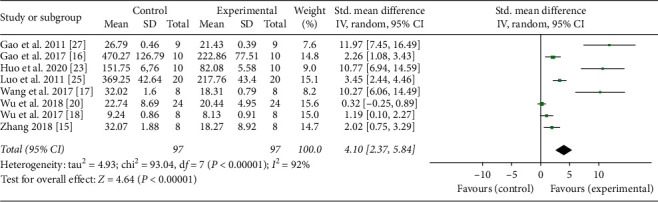
Forest plot showed that the level of IL-1*β* decreased with moxibustion therapy.

**Figure 4:**
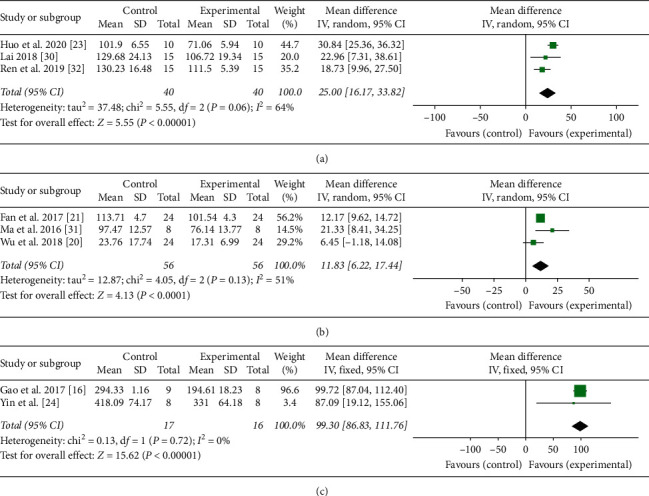
Forest plot showed that the level of proinflammatory cytokines decreased with moxibustion therapy. (a), (b), and (c), respectively, represented the effect of moxibustion on IFN-*γ*, IL-6, and IL-17.

Eleven studies adopted TNF-*α* as an outcome index [15–25]. The pooled results showed that moxibustion significantly decreased the level of TNF-*α* compared with the control group (*n* = 249, SMD 2.95, 95% CI: 1.99–3.92, *P* < 0.00001; heterogeneity *X*^2^ = 63.44, *I*^2^ = 84%, Figure 2). IL-1*β* was reported as outcome in eight studies [15–18, 23, 25–27]. The result showed that moxibustion reduced the level of IL-1*β* compared with the control group (*n* = 193, SMD 4.10, 95% CI: 2.37–5.84, *P* < 0.00001; heterogeneity *X*^2^ = 93.04, *I*^2^ = 92%, Figure 3). Three studies regarded IFN-*γ* as an outcome index [23, 30, 32] and the result showed that IFN-*γ* was significantly different between the moxibustion group and the control group (*n* = 80, MD 25.0, 95% CI: 16.17–33.82, *P* < 0.00001; heterogeneity *X*^2^ = 5.55, *I*^2^ = 64%, Figure 4(a)). IL-6 was used as an outcome indicator in three studies [21, 26, 31]. The pooled results showed that moxibustion significantly decreased IL-6 (*n* = 116, MD 11.83, 95% CI: 6.22–17.44, *P* < 0.00001; heterogeneity *X*^2^ = 4.05, *I*^2^ = 51%, Figure 4(b)). Pooled results of two studies showed that the content of IL-17 was lower in the moxibustion group (*n* = 37, MD 99.3, 95% CI: 86.83–111.76, *P* < 0.00001; heterogeneity *X*^2^ = 0.13, *I*^2^ = 0%, Figure 4(c)).

#### 3.6.2. Anti-Inflammatory Cytokines Outcomes Analysis

**Figure 5:**
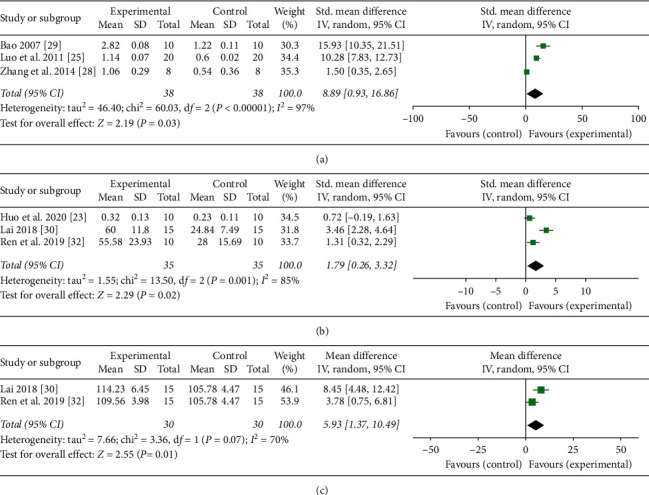
Forest plot showed that the level of anti-inflammatory cytokines increased with moxibustion therapy. (a), (b), and (c), respectively, represented the effect of moxibustion on IL-2, IL-4, and IL-10.

Three studies were conducted pooled analysis of IL-2 [25, 28, 29]. The pooled results showed that moxibustion significantly increased the level of IL-2 (*n* = 76, SMD 8.89, 95% CI: 0.93–16.86, *P*=0.03; heterogeneity *X*^2^ = 60.03, *I*^2^ = 97%, Figure 5(a)). The pooled results of other three studies [23, 27, 30] showed that the moxibustion had an effect on IL-4 with a higher expression compared with control groups (*n* = 70, MD 1.79, 95% CI: 0.26–3.32, *P*=0.02; heterogeneity *X*^2^ = 13.5, *I*^2^ = 85%, Figure 5(b)). Two studies [30, 32] reported that the moxibustion increased the level of IL-10 compared with the control group (*n* = 60, MD 5.93, 95% CI: 1.37–10.49, *P*=0.01; heterogeneity *X*^2^ = 3.36, *I*^2^ = 70%, Figure 5(c)).

### 3.7. Subgroup Analyses

#### 3.7.1. TNF-*α*

The researchers performed a subgroup analysis for the moxibustion methods. The result showed that suspension moxibustion had a significant effect on the reduction of the level of TNF-*α* (SMD 2.36, 95% CI: 1.51–3.22; *P* < 0.00001), but no remarkable difference was found in the seed-sized moxa cone (SMD 5.20, 95% CI: −0.25–10.66; *P*=0.06). Subgroup analysis of the moxibustion methods indicated that suspension moxibustion reduced heterogeneity (*I*^2^ = 68%), while seed-sized moxa cone did not (*I*^2^ = 94%). Moxibustion was found to have a remarkable effect on the reduction of TNF-*α* in both Sprague-Dawley rats (SMD 2.96, 95% CI: 1.62–4.30; *P* < 0.0001) and Wister rats (SMD 3.17, 95% CI: 1.37–4.98; *P*=0.0006). The present study also conducted a subgroup analysis for the modeling methods and found that CFA model (SMD 2.83, 95% CI: 1.55–4.12; *P* < 0.0001) and CIA model (SMD 2.37, 95% CI: 1.05–3.69; *P*=0.0004) performed well in the reduction of the level of TNF-*α*. In the subgroup analysis, the reviewers compared the effects of different genders on TNT-*α*. The declining level of TNF-*α* in male rats (SMD 3.19, 95% CI: 1.97–4.41; *P* < 0.00001) was higher than that of the male–female experiment (SMD 2.59, 95% CI: 0.38–4.80; *P*=0.02). The researchers also compared the effects of different body weights on TNF-*α* in subgroups. The results showed that rats weighing from 180 to 220 g (SMD 3.34, 95% CI: 1.90–4.79; *P* < 0.00001) had significantly lower levels of TNF-*α*, while rats weighing from 110 to 150 g (SMD 4.82, 95% CI: −1.24–10.87; *P*=0.12) and 160 to 200 g (SMD 2.16, 95% CI: −0.72–5.04; *P*=0.15) had no significant effect on TNF-*α* levels. Compared with unpublished articles, published articles showed more obvious changes on TNF-*α* with moxibustion treatment (SMD 2.80, 95% CI: 1.79–3.81; *P* < 0.00001). A summary of the subgroup analysis is shown in Table 3.

#### 3.7.2. IL-1*β*

In the subgroup analysis of IL-1*β*, the efficacy of suspension moxibustion (SMD 4.58, 95% CI: 2.30–6.86, *P* < 0.0001) was better than that of the seed-sized moxa cone (SMD 3.60, 95% CI: 0.49–6.71, *P*=0.02). In regard to the varieties of experimental animals, SD rats (SMD 3.25, 95% CI: 1.47–5.04, *P*=0.0004) were more sensitive to moxibustion than Wister rats (SMD 6.87, 95% CI: −0.29–14.02, *P*=0.06). By analyzing different causes of RA models, we found that CFA models (SMD 4.75, 95% CI: 2.19–7.30, *P*=0.0003) performed better than CIA models (SMD 3.47, 95% CI: 0.67–6.27, *P*=0.02) in decreasing IL-1*β*. The study found that IL-1*β* levels were significantly reduced in experiments using male rats (SMD 3.89, 95% CI: 2.14–5.65, *P* < 0.0001), but there was no statistically significant difference in experiments using male–female rats (SMD 5.36, 95% CI: −4.87–15.60, *P*=0.3). Rats with different weights were included in these studies. The reviewers found that rats with weight ranging from 180 to 220 g (SMD 5.71, 95% CI: 2.49–8.94, *P* < 0.0001) were more sensitive to moxibustion for the improvement of IL-1*β* than rats with weight ranging from 160 to 200 g (SMD 4.17, 95% CI: 0.72–7.62, *P*=0.02). Compared with unpublished articles, published articles showed more obvious changes on IL-1*β* with moxibustion treatment (SMD 4.61, 95% CI 2.58–6.64; *P* < 0.0001). A summary of the subgroup analysis is shown in Table 4.

### 3.8. Biological Mechanism

Several different biological mechanisms were studied to better understand the potential mechanism of the anti-inflammatory effects of moxibustion on the treatment of RA. Among the eighteen studies, fourteen got detailed descriptions about the possible mechanisms. It was found that the main biological mechanisms were categorized into three aspects: regulation of the hypothalamic-pituitary-adrenal axis (HPAA), restoration of the balance between T helper 1 cell (Th1) and T helper 2 cell (Th2), and regulation of the programmed cell death 1 and ligand 1 (PD-1/PD-L1). The summary of the proposed mechanisms is shown in Table 5.

### 3.9. Assessment of Bias

**Figure 6:**
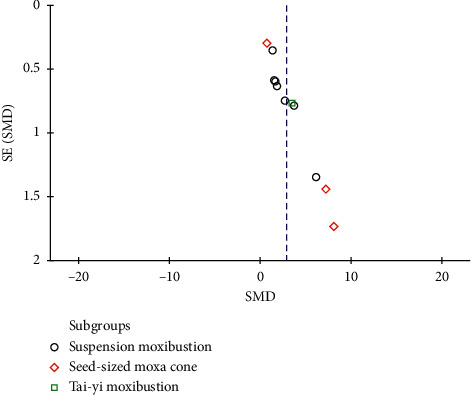
Funnel plot for the effectiveness of moxibustion on TNF-*α*.

Funnel plot was applied to assess the publication bias. The asymmetry funnel plot indicated that there was publication bias (Figure 6).

## 4. Discussion

### 4.1. Principle Findings

The current study represents the first meta-analysis to evaluate the effect of moxibustion for the treatment of RA in animal experiments with the results of inflammatory cytokines as outcome indicators. This meta-analysis indicated that moxibustion has certain effects on the amelioration of the inflammation in animal models with RA, including the downregulation of the level of proinflammatory cytokines and the upregulation of the level of anti-inflammatory cytokines at the same time. Clinical trials revealed that moxibustion can enhance the anti-inflammatory and analgesic effects of conventional medicine and downregulate HIF-1*α*/VEGF contents to inhibit angiogenesis [33]. The conclusion is similar to the previous clinical meta-analysis [34].

### 4.2. Possible Therapeutic Mechanism of the TCM

“The Yellow Emperor's Internal Classic,” an ancient medical canon of TCM in China, holds that the occurrence of RA is closely related to the invasion of three perverse trends of wind, cold, and dampness. The primary pathogenesis is the deficiency of healthy Qi in viscera and the meridian is blocked by accumulated dampness, which has close relationship with spleen, stomach, and kidney. Therefore, warming the meridian and removing dampness through invigorating spleen and kidney are the basic principles of treatment [35]. Almost all the selected acupoints belong to the Stomach Meridian and Kidney Meridian. It is consistent with the basic pathogenesis of the Qi deficiency and pathogenic stagnation, which plays a key role in ensuring effectiveness by warming the meridian and eliminating dampness through invigorating spleen and kidney, dredging channel of Qi and blood, and harmonizing Yin and Yang.

The theory of TCM holds that spleen and stomach provide the material basis of the acquired constitution. ST 36, the he-sea point of Stomach Meridian of Foot-Yangming, can strengthen the function of spleen and stomach and eliminate dampness. The results of previous studies indicated that acupuncture at ST 36 can promote gastrointestinal motility and improve gastrointestinal function [36, 37]. The research found that electroacupuncture at ST 36 might activate AMPK, produce stable ULK1/AMPK compound, and increase the mitochondrial autophagy, which could regulate spleen-stomach and treat spleen deficiency [38]. Meanwhile, acupuncture at ST 36 reduced the lipopolysaccharide- (LPS-) induced serum levels of TNF, IL-6, and INF-*γ* [39] and could promote eosinophils apoptosis to inhibit the development of inflammatory reaction [40]. BL23 is the back-shu point of kidney. Moxibustion at BL23 for the treatment of RA can not only nourish kidney and benefit essence, but also dispel cold and relieve pain, so as to achieve the effect of treating both symptoms and root causes.

### 4.3. Possible Modern Biological Mechanism

The combination of the previously mentioned analysis and numerous research results indicates that the biological mechanism of moxibustion in the treatment of RA involves several aspects. Researchers have conducted many studies from the perspective of anti-inflammatory, immune regulation, regulation of circadian rhythm, and bone protection [41–44]. Among them, there are studies concentrating on anti-inflammatory effect as the most important aspect because RA is characterized by synovitis, which may result in cartilage and bone destruction. Previous studies explored the mechanisms of moxibustion in treating RA from several aspects, including restoring the balance between Th1 and Th2 by regulating the levels of inflammatory cytokines [23, 27], modulating the pathological circadian rhythm of TNF-*α*, IL-1*β*, and IL-6 [20, 25, 31], regulating the function of HPAA [31], alleviating cartilage degradation through RANKL/OPG signaling pathway [44], and strengthening the negative regulation of PD-1/PD-L1 signaling pathway [30, 32]. This study suggested that moxibustion can relieve synovitis of the joint with RA mainly by inhibiting the secretion of proinflammatory cytokines and enhancing the secretion of anti-inflammatory cytokines to restore the equilibrium between Th1 and Th2. Moxibustion is effective in treating RA, but its mechanism of action involves many aspects, and its anti-inflammatory effect is just one of them. Therefore, it needs to be further explored in future studies.

### 4.4. The Value of Animal Studies and Systematic Review of the Effectiveness of Moxibustion for RA in Animal Model

As a complementary therapy, moxibustion is safe and effective for people suffering from RA to some extent. However, there is also a controversial view that moxibustion has no therapeutic effect but only a warming effect. Revealing the mechanisms of moxibustion for RA and making sure that the effect is not just warming are significant for the development of moxibustion. To dispel these doubts, it is necessary to objectively investigate the effects of moxibustion on synovium and related tissues, cells, and signal pathways. As is known to all, it is unethical and forbidden to obtain tissues or cells from human body. For this reason, animal researches of moxibustion for RA are of great value and cannot be replaced completely by clinical studies. Meta-analysis is a kind of statistical method that can comprehensively analyze the results of many studies and help researchers promote the methodological quality of preclinical animal researches. Therefore, we conducted this systematic review and meta-analysis to assess the effectiveness of moxibustion in the treatment of RA in animal models and found that moxibustion has a definite effect to relieve inflammation in animal models with RA.

### 4.5. Subgroup Analysis

The high heterogeneity among the studies based on inflammation cytokines cannot be completely explained. The reviewers carried out subgroup analysis on the included studies based on moxibustion methods, modeling methods, animal species, animal gender, and animal weight, and the researchers also conducted sensitivity analysis, but no evident cause was found. However, in subgroup analysis, it was found that suspended moxibustion was significantly better than seed-sized moxa cone in improving the level of inflammatory cytokines. The reviewers also found that 13 of the 18 studies used ST36 and BL23 points, and the remaining five studies involved acupoints including CV4, GV4, GB34, SP9, and behind ST36 or a combination of the previously mentioned points. The acupoints used were inconsistent. Furthermore, the sample size also covered a wide scope ranging from 16 to 48 and no study mentioned sample size calculation. These findings suggested that, in addition to the differences in moxibustion method, modeling methods and animal species, acupoint combinations, and sample sizes may be another source of heterogeneity among the studies.

### 4.6. Limitations and Strengths

The present review has several limitations. First, it is the language. The researchers limited the languages of all studies to English and Chinese. Of all the included studies, only one was written in English, which may cause potential publication bias. Second, the scope of sample size still needs to be expanded. Although the researchers conducted a comprehensive literature search and traced the references of the included literature to supplement the relevant literature, no literatures met the inclusion criteria. Third, the quality of the included studies is unsatisfactory and the differences in the selection of acupoints and operation methods may greatly affect the outcome of the meta-analysis. Finally, the blind method was not performed well. Although it is difficult to blind moxibustion for operators, it should be strictly carried out in the process of molding and the assessment of outcome.

According to the above limitations, more databases with different languages should be searched to increase the diversity of the included literature. Meanwhile, in terms of experimental design, especially the random allocation of treatment and control, blind building of model and blind assessment of outcome should be strictly carried out in future animal researches. In addition, a unified standard of moxibustion therapeutic schedule should be seriously performed because the details of moxibustion manipulation are closely related to the efficacy. The choice of moxibustion methods and moxibustion acupoints, duration of each moxibustion, and course of treatment are necessarily to be standardized.

## 5. Conclusions

The findings of the present review indicate that moxibustion can protect the synovium of joint in animal models with RA by enhancing the level of anti-inflammatory cytokines and decreasing the level of proinflammatory cytokines. Moxibustion has the potential to relieve inflammation in animal models with RA. However, the specific mechanism still needs to be further explored and the quality of studies should also be improved in the future. Therefore, future researches need to be more standardized in terms of experimental design, especially the design of blind method, selection of inflammatory cytokines, and moxibustion methods so as to provide a reliable theoretical basis for the application of basic research achievements to clinical practice.

## Figures and Tables

**Table 1:** Characteristics of the 18 included studies.

Study	Species (*Nc*/*Nm*)	Gender	Age (week)	Weight (g)	Model	Moxibustin (acupoints)	Control	Duration (day)	Outcome index	*P* value
Zhang 2018 [15]	SD rat (8/8)	Male	NR	200–250	CIA	SM (BL23, ST36)	No treatment	21	TNF-*α*;	*P* < 0.05
									IL-1*β*	*P* < 0.05
Gao et al. 2017 [16]	SD rat (8/9)	Male	Adult	200 ± 20	CFA	SSMC (BL23, ST36)	No treatment	21	TNF-*α*;	*P* < 0.01
									IL-1*β*;	*P* < 0.01
									IL-17	*P* < 0.01
Wang et al. 2017 [17]	SD rat (8/8)	Male	NR	200 ± 20	CIA	SM (BL23, ST36)	No treatment	21	TNF-*α*;	*P* < 0.01
									IL-1*β*	*P* < 0.01
Wu et al. 2017 [18]	SD rat (8/8)	Male	7-8	NR	CIA	SM (BL23, ST36)	No treatment	21	TNF-*α*;	*P* < 0.05
									IL-1*β*	*P* < 0.05
Su et al. 2015 [19]	SD rat (10/10)	Male/female	8–10	200 ± 20	CFA	TYM governor meridian	No treatment	12	TNF-*α*	*P* < 0.05
Wu et al. 2018 [20]	SD rat (24/24)	Male/female	Adult	180 ± 20	CFA	SSMC (BL23, ST36)	No treatment	21	TNF-*α*	*P* < 0.01
Fan et al. 2017 [21]	SD rat (10/10)	Male	NR	200 ± 20	CFA	SM (BL23, ST36, GB34)	No treatment	21	TNF-*α*;	*P* < 0.01
									IL-6	*P* < 0.01
Cui 2019 [22]	Wistar rat (8/8)	Male	NR	140 ± 15	CIA + CFA	SSMC (BL23, ST36, SP9, GB34)	No treatment	20	TNF-*α*	*P* < 0.05
Huo et al. 2020 [23]	Wistar rat (10/10)	Male/female	NR	180 ± 20	CFA	SM (BL23, ST36)	No treatment	21	TNF-*α*;	*P* < 0.05
									IL-1*β*;	*P* < 0.01
									IFN-*γ*;	*P* < 0.05
									IL-4	*P* < 0.01
Yin et al. 2017 [24]	Wistar rat (10/10)	Male	NR	125 ± 15	CIA	SM (BL23, ST36)	No treatment	16	TNF-*α*;	*P* < 0.05
									IL-17	*P* < 0.05
Luo et al. 2011 [25]	Wistar rat (20/20)	Male	12–16	200 ± 20	CFA	SM (BL23, ST36)	No treatment	15	TNF-*α*;	*P* < 0.01
									IL-1*β*;	*P* < 0.01
									IL-2	*P* < 0.01
Wu et al. 2018 [26]	SD rat (24/24)	Male/female	Adult	200 ± 20	CFA	SSMC (BL23, ST36)	No treatment	21	IL-1*β*;	*P* < 0.05
									IL-6	*P* < 0.05
Gao et al. 2011 [27]	SD rat (10/10)	Male	NR	200 ± 20	CFA	SSMC (BL23, ST36)	No treatment	21	IL-1*β*;	*P* < 0.01
									IL-4	*P* < 0.05
Zhang et al. 2014 [28]	Wistar rat (8/8)	Male	12–16	160–180	CFA	SSMC (BL23, behind BL23)	No treatment	20	IL-2	*P* < 0.01
Bao 2007 [29]	SD rat (10/10)	Male/female	NR	200 ± 20	CFA	TM (CV4, GV4)	No treatment	25	IL-2	*P* < 0.01
Lai 2018 [30]	SD rat (15/15)	Male/female	Adult	200 ± 20	CFA	SSMC (BL23, ST36)	No treatment	21	IL-4;	*P* < 0.01
									IL-10;	*P* < 0.05
									IFN-*γ*	*P* < 0.01
Ma et al. 2016 [31]	SD rat (24/24)	Male/female	Adult	200 ± 20	CFA	SSMC (BL23, ST36)	No treatment	21	IL-6	*P* < 0.05
Ren et al. 2019 [32]	SD rat (15/15)	Male/female	NR	200 ± 20	CFA	SSMC (BL23, ST36)	No treatment	21	IFN-*γ*;	*P* < 0.05
									IL-10	*P* < 0.05

*Nm*: number of animals in the moxibustion group, *Nc*: number of animals in the control group, SD sprague dawley, *NR*: no record, *CIA*: collagen-induced arthritis, *CFA*: complete Freund's adjuvant, *SM*: suspension moxibustion, *SSMC*: seed-sized moxa cone, and *TYM*: tai-yi moxibustion.

**Table 2:** Methodological quality assessment of the studies included.

Study	①	②	③	④	⑤	⑥	⑦	⑧	⑨	⑩	Total
Zhang 2018 [15]			√				√				2
Gao et al. 2017 [16]	√		√			√	√				4
Wang et al. 2017 [17]	√	√	√	√		√	√		√	√	8
Wu et al. 2017 [18]	√	√	√	√			√		√	√	7
Su et al. 2015 [19]	√		√				√				3
Wu et al. 2018 [20]	√	√	√		√	√	√		√	√	8
Fan et al. 2017 [21]	√		√				√			√	4
Cui 2019 [22]			√			√	√		√		4
Huo et al. 2020 [23]	√	√	√	√		√	√		√	√	8
Yin et al. 2017 [24]	√	√	√	√			√		√	√	7
Luo et al. 2011 [25]	√	√	√			√	√		√	√	7
Wu et al. 2018 [26]	√	√	√		√	√	√		√	√	8
Gao et al. 2011 [27]	√		√				√			√	4
Zhang et al. 2014 [28]	√	√	√			√	√		√	√	7
Bao 2007 [29]		√	√				√				3
Lai 2018 [30]			√		√	√	√				4
Ma et al. 2016 [31]	√	√	√		√	√	√		√	√	8
Ren et al. 2019 [32]	√	√	√		√	√	√		√	√	8

①, peer-reviewed publication; ②, control of temperature; ③, random allocation to treatment or control; ④, blinded induction of model; ⑤, blinded assessment of outcome; ⑥, use of anesthetic without significant intrinsic neuroprotective activity; ⑦, appropriate animal model; ⑧, sample size calculation; ⑨, compliance with animal welfare regulations; ⑩, statement of potential conflict of interests.

**Table 3:** Subgroup analysis for the effect of moxibustion on the reduction of TNF-*α*.

		Heterogeneity		Effect size	
		*P*	*I* ^2^	95% CI	*P*
Moxibustion	SS	0.005	68	SMD = 2.36 (1.51, 3.22)	<0.00001
	SSMC	0.00001	94	SMD = 5.20 (−0.25, 10.66)	=0.06

Model	CFA	0.00001	87	SMD = 2.83 (1.55, 4.12)	<0.0001
	CIA	0.02	71	SMD = 2.37 (1.05, 3.69)	=0.0004

Species	SD	0.00001	86	SMD = 2.96 (1.62, 4.30)	<0.0001
	Wister	0.0002	85	SMD = 3.17 (1.37, 4.98)	=0.0006

Weight	110–155 g	0.0001	91	SMD = 4.82 (−1.24, 10.87)	=0.12
	160–200 g	0.0005	92	SMD = 2.16 (−0.72, 5.04)	=0.15
	180–220 g	0.0001	83	SMD = 3.34 (1.90, 4.79)	<0.00001

Gender	Male	0.00001	82	SMD = 3.19 (1.97, 4.41)	<0.00001
	Male/female	0.0001	90	SMD = 2.59 (0.38, 4.80)	=0.02

Publication	Yes	0.00001	84	SMD = 2.80 (1.79, 3.81)	<0.00001
	No	0.0003	92	SMD = 4.64 (−1.76, 11.05)	=0.16

**Table 4:** Subgroup analysis for the effect of moxibustion on reducing IL-1*β*.

		Heterogeneity		Effect size	
		*P*	*I* ^2^ (%)	95% CI	*P*
Moxibustion	SM	0.00001	90	SMD = 4.58 (2.30, 6.86)	<0.0001
	SSMC	0.00001	94	SMD = 3.60 (0.49, 6.71)	=0.02

Model	CFA	0.00001	95	SMD = 4.75 (2.19, 7.30)	= 0.0003
	CIA	0.0002	88	SMD = 3.47 (0.67, 6.27)	=0.02

Species	SD	0.00001	91	SMD = 3.25 (1.47, 5.04)	=0.0004
	Wister	0.0003	92	SMD = 6.87 (−0.29, 14.02)	=0.06

Weight	160–200 g	0.00001	96	SMD = 4.17 (0.72, 7.62)	=0.02
	180–220 g	0.00001	90	SMD = 5.71 (2.49, 8.94)	<0.00001

Gender	Male	0.00001	87	SMD = 3.89 (2.14, 5.65)	<0.0001
	Male/female	0.00001	96	SMD = 5.36 (−4.87, 15.60)	=0.3

Publication	Yes	0.00001	94	SMD = 4.61 (2.58, 6.64)	<0.00001
	No			SMD = 2.02 (0.75, 3.29)	=0.002

**Table 5:** Summary of the proposed mechanisms.

Study	Findings or proposed mechanisms
Zhang 2018 [15]	Decreased TNF-*α*, IL-1*β*, PGE_2_
	Adjust abnormal levels of metabolites
Gao et al. 2017 [16]	Reduce TNF-*α*, IL-1*β*, IL-17
Wang et al. 2017 [17]	Regulated the immune function
Wu et al. 2017 [18]	Decreased TNF-*α*, IL-1*β*
Su et al. 2015 [19]	Reduced T cell activation
	Regulated the immune function
Wu et al. 2018 [20]	Regulated the circadian rhythm of TNF-*α*
Fan et al. 2017 [21]	Decreased TNF-*α*, IL-6, NO, TNF-*α* mRNA
Cui 2019 [22]	Reduced TNF-*α*, BK
Huo et al. 2020 [23]	Decrease TNF-*α*, IFN-*γ*, IL-1*β*, IL-4
	Restore the balance between Th1/Th2
Yin et al. 2017 [24]	Decrease TNF-*α*, IL-17
Luo et al. 2011 [25]	Increased IL-2
	Decreased TNF-*α*, IL-1*β*
Wu et al. 2018 [26]	Rhythmic anti-inflammatory
Gao et al. 2011 [27]	Increased IL-4
	Decreased IL-1*β*, MT
	Restore the balance between Th1/Th2
Zhang et al. 2014 [28]	Increased IL-2, Bcl-2
	Decreased IL-1, caspase-3
Bao 2007 [29]	Regulated the immune function
Lai 2018 [30]	Inhibit the activation of T cell
	Regulate the PD-1/PD-L1 signaling pathway
Ma et al. 2016 [31]	Regulated the circadian rhythm of IL-6
	Regulate the HPAA
Ren et al. 2019 [32]	Regulate the PD-1/PD-L1 signaling pathway

*PGE*_*2*_: prostaglandin *E*_2_, *NO*: nitric oxide, *BK*: bradykinin, *MT*: melatonin, *Th1*: T helper 1 cell, *Th2*: T helper 2 cell, *HPAA*: the hypothalamic-pituitary-adrenal axis, *Bcl-2*: B-cell lymphoma-2, *PD-1*: programmed cell death 1, *PD-L1*: programmed cell death ligand 1.
